# Case Report: Syndrome of inappropriate antidiuretic hormone secretion induced by pramipexole: a case report and literature review

**DOI:** 10.3389/fphar.2025.1627245

**Published:** 2025-08-15

**Authors:** Ke-Qian Chen, Xiang Liu, Hai-Bo Lei

**Affiliations:** Department of Clinical Pharmacy, Xiangtan Central Hospital (The Affiliated Hospital of Hunan University), Xiangtan, China

**Keywords:** pramipexole, syndrome of inappropriate antidiuretic hormone secretion, adverse drug reactions, ADH, SIADH

## Abstract

Pramipexole, a novel dopamine receptor agonist, is widely used in the treatment of Parkinson’s disease and related syndromes. While studies have demonstrated that pramipexole can cause various adverse reactions, primarily involving the central nervous system or gastrointestinal symptoms, there remains limited reporting on pramipexole-induced syndrome of inappropriate antidiuretic hormone secretion (SIADH). This article analyzes and discusses a case of pramipexole-induced SIADH that occurred in our hospital. At the same time, we also summarize and discuss all current cases of pramipexole-induced SIADH. We anticipate these findings will serve as a valuable clinical reference to guide appropriate medication use.

## Introduction

Parkinson’s disease (PD) is a neurological disorder with evolving layers of complexity. It has long been characterised by the classical motor features of parkinsonism associated with Lewy bodies and loss of dopaminergic neurons in the substantia nigra. However, the symptomatology of PD is now recognised as heterogeneous, with clinically significant non-motor features. Similarly, its pathology involves extensive regions of the nervous system, various neurotransmitters, and protein aggregates other than just Lewy bodies (Kalia and Lang, 2015). Pramipexole is a dopamine receptor agonist and has a high affinity for D2, D3, and D4 receptors. On the one hand, pramipexole is primarily used for the treatment of early-onset PD. Meanwhile, it is also used in the treatment of treatment-resistant depression, the treatment of anhedonia, or bipolar depression (Lemke et al., 2006). On the other hand, pramipexole can be used alone or in combination with levodopa to treat advanced PD. While studies have demonstrated that pramipexole can cause various adverse reactions, primarily involving the central nervous system or gastrointestinal symptoms (Xu et al., 2012), there remains limited reporting on pramipexole-induced syndrome of inappropriate antidiuretic hormone secretion (SIADH). SIADH is a syndrome characterized by clinical manifestations such as water retention in the body, increased urinary sodium excretion, and hyponatremia. The essential diagnostic criteria for hyponatremia secondary to SIADH include Serum sodium<135 mmol/L, Decreased measured plasma osmolality<275 mOsm/kg, Urinary osmolality >100 mOsm/kg during hypotonicity, Clinical euvolemia (No clinical signs of contraction of extracellular fluid, No clinical signs of expansion extracellular fluid), Increased urinary sodium excretion >30 mmol/L with normal dietary salt and water intake, Normal thyroid and adrenal function determined by both clinical and laboratory assessment, No recent use of diuretic agents. The supporting diagnostic criteria for hyponatremia secondary to SIADH include Plasma uric acid <4 mg/dL (<0.24 mmol/L), Blood urea nitrogen <10 mg/dL (<3.6 mmol/L), Fractional sodium excretion >1%, fractional urea excretion >55%, Failure to improve hyponatremia after 0.9% saline infusion, Improvement of hyponatremia with fluid restriction. The typical symptoms of hyponatremia include weakness, fatigue, nausea, vomiting, confusion and muscle spasms (Zhou and Wang, 2014). It is mainly caused by various reasons such as malignant tumors, lung diseases, central nervous system diseases, and drugs (such as SSRI, SNRI, proton pump inhibitors, thiazide and loop diuretics, carbamazepine, *etc.*), resulting in an abnormal increase of endogenous antidiuretic hormone (ADH) secretion (Mo et al., 2025). Meanwhile, a very important risk factor for hyponatremia in the course of pharmacotherapy is also old age (Pisa and Del Cerro, 2019). This article analyzes and discusses a case of pramipexole-induced SIADH that occurred in our hospital. At the same time, we also summarize and discuss all current cases of pramipexole-induced SIADH (Chen et al., 2019; Zhang and Li, 2024; Choi et al., 2010; Tomita et al., 2005; Arai and Iwabuchi, 2009) (Table 1). We anticipate these findings will serve as a valuable clinical reference to guide appropriate medication use.

**TABLE 1:** Cases of pramipexole-induced SIADH.

Age	Gender	Diagnose	Relevant examination	Treatment	ADR time	Country	Results	Exposure	References
69	Female	PD	Thyroid Adrenal gland	Levodopa/Carbidopa + Entacapone + Pramipexole	\	China	After stopping pramipexole, the symptom relieved rapidly and the blood sodium returned to normal	\	Chen et al. (2019)
78	Male	PD	Thyroid Brain Chest	Levodopa and benserazide + Pramipexole	180 days after taking pramipexole	China	The patient discontinued pramipexole and was treated with 10% sodium chloride. After 7 days, the blood sodium returned to normal	No	Zhang and Li (2024)
75	Female	PD	Thyroid Adrenal gland	Amantadine + Pramipexole + Glimepiride + alprazolam + aspirin + Stalevo + Sertraline + Escitalopram + Carbidopa/levodopa + Domperidone + Quetiapine + Propranolol + Donepezil + Clonazepam + Warfarin + Amlodipine + Losartan	111 days after taking pramipexole	Korea	After stopping pramipexole, the symptom relieved rapidly and the blood sodium returned to normal on the 13th day	No	Choi et al. (2010)
85	Female	PD	Thyroid Brain Adrenal gland	Levodopa/Carbidopa + Pramipexole	7 days after taking pramipexole	Japan	The patient discontinued pramipexole and was treated with 3 g sodium chloride. After 7 days, the blood sodium returned to normal	\	Tomita et al. (2005)
60	Male	PD	Thyroid Adrenal gland	Levodopa/Carbidopa + Entacapone + Selegiline +Pramipexole+ Cabergoline + Pergolide	270 days after taking pramipexole	Japan	The patient reduced the dose of pramipexole. After 30 days, the blood sodium returned to normal	Yes	Arai and Iwabuchi (2009)

## Clinical presentation

An 84-year-old woman visited our neurology outpatient clinic duo to dizziness, fatigue, and limb tremors. Upon neurological examination, she had bradykinesia and intermittent limb tremors. This patient has a history of percutaneous coronary intervention (PCI) surgery for coronary heart disease, pituitary tumor surgery, type 2 diabetes with peripheral neuropathy and retinopathy, and hypertension. Her current medications include isophane protamine human insulin (21U sc qd), metformin hydrochloride extended-release tablets (0.5 g po qd), and indapamide tablets (2.5 mg po qd) for blood pressure and blood sugar control. The patient denies a history of infectious diseases such as hepatitis, typhoid fever, and tuberculosis. Additionally, this patient has a known allergy to ceftazidime.

The patient was hospitalized on July 28th (Day 1). Cranial CT revealed senile brain atrophy, white matter degeneration, and multiple lacunar cerebral infarctions. Biochemical studies showed the following electrolyte levels: Serum sodium: 139 mmol/L, Serum potassium: 4.04 mmol/L, and Serum chloride: 99 mmol/L. On July 30th (Day 3), the patient was diagnosed with Parkinson’s syndrome. Electromyography detected a 4.0 Hz tremor in both upper limbs at rest. The patient exhibited Parkinson’s motor symptoms, including bradykinesia, muscle rigidity, and tremor, as well as non-motor symptoms such as dizziness, constipation, sleep disorders, anxiety, and depression. Treatment was initiated with oral dopashydrazine tablets (125 mg po tid) and pramipexole tablets (0.125 mg po tid). On August 5th (Day 5), the patient developed dizziness, drowsiness, and vomiting. Biochemical studies revealed significant electrolyte imbalances: Serum sodium: 114.2 mmol/L, Serum potassium: 3.35 mmol/L, Serum chloride: 74.3 mmol/L, Effective plasma osmotic pressure: 249.8 mmol/L (<275 mmol/L). Cortisol and adrenocorticotropic hormone levels were within normal ranges. The diagnosis was hypotonic hyponatremia, which was attributed to pramipexole-induced SIADH after consultation with the clinical pharmacy and endocrinology departments. The patient’s blood volume status was euvolemia, with oliguria and high urinary osmolarity. Brain and chest CT scans showed no abnormalities, and pituitary, adrenal, and thyroid functions (corticotropin: 8.16 pg/mL, thyrotropin: 2.34 µIU/mL) were normal. SIADH caused by underlying conditions (malignancies, lung diseases, central nervous system diseases) was ruled out. Pramipexole was discontinued, and the patient was treated with 10% sodium chloride injection (20 mL po bid) and potassium chloride sustained-release tablets (1 g po bid). On August 7th (Day 11), biochemical studies showed the following electrolyte levels: Serum sodium: 137 mmol/L, Serum potassium: 3.9 mmol/L, Serum chloride: 104 mmol/L. On August 11th (Day 15), biochemical studies showed the following electrolyte levels: Serum sodium: 136 mmol/L, Serum potassium: 4.39 mmol/L, Serum chloride: 103 mmol/L.

## Literature review

This study utilized the CNKI, Web of Science, and PubMed databases to search for case reports related to “pramipexole” and “antidiuretic hormone” published between 2000 and 2024. Through systematic screening, we identified five reported cases of pramipexole-induced SIADH. Including the present case, a total of six cases (China: 3 cases, South Korea: 1 case, Japan: 2 cases) have been documented worldwide. The study included 6 patients (4 females and 2 males) aged 60–85 years. The onset of SIADH typically occurred between 2 and 270 days after initiating pramipexole treatment. Symptom resolution was achieved through pramipexole dose reduction or discontinuation, fluid restriction, and hypertonic saline administration. Serum sodium levels returned to normal within 30 days of dose reduction or within 7–13 days after complete discontinuation. Among the 6 patients, 3 patients had no re-exposure to pramipexole, 2 patients lacked documented re-exposure status, and 1 patient maintained treatment at a reduced dose without SIADH recurrence.

## Discussion

According to national adverse drug reaction monitoring principles, this case was analyzed as follows: Both the package insert and literature suggest that pramipexole can cause SIADH. The patient’s electrolyte levels were normal prior to medication but developed hyponatremia and hypochloremia following pramipexole initiation, accompanied by anorexia, vomiting, and mental status deterioration. After discontinuing pramipexole and administering hypertonic saline treatment, electrolyte levels normalized within 2 days. Subsequent monitoring showed no recurrence of electrolyte abnormalities without pramipexole rechallenge. Brain and chest CT scans showed no abnormalities, and pituitary, adrenal, and thyroid functions were normal. SIADH caused by underlying conditions (malignancies, lung diseases, central nervous system diseases) was ruled out. These findings establish a “very likely” causal relationship between pramipexole and SIADH in this case.

The mechanism of pramipexole-induced SIADH is as follows:Pramipexole is a dopamine receptor agonist and has a high affinity for D2, D3, and D4 receptors (Azdad et al., 2003). ADH, synthesized by neurons in the hypothalamic supraoptic and paraventricular nuclei, is regulated by γ-aminobutyric acid (GABA). The D4 receptors are located at the GABA terminal within the supraoptic nucleus. By activating D4 receptors, pramipexole reduces GABA release, which in turn promotes ADH secretion (Kvernmo et al., 2006). This neuroendocrine disruption leads to increased water reabsorption, ultimately resulting in SIADH.

SIADH may present with symptoms including nausea, vomiting, weakness, drowsiness, and mental confusion. The therapeutic approach involves: correcting underlying etiologies, implementing fluid restriction, administering intravenous sodium chloride solution, and utilizing ADH receptor antagonists or diuretics. Treatment plan should be tailored to individual patient conditions. Drug-induced SIADH require immediate drug withdrawal, and low serum sodium levels can be corrected after drug withdrawal (Cuesta et al., 2016). Therefore, PD patients initiating pramipexole therapy require regular serum sodium monitoring to facilitate early detection and management of drug-induced SIADH.

## Data Availability

The original contributions presented in the study are included in the article/supplementary material, further inquiries can be directed to the corresponding authors.
